# Leading the next pandemics

**DOI:** 10.1016/j.puhip.2025.100605

**Published:** 2025-03-20

**Authors:** Obinna O. Oleribe

**Affiliations:** California State University, Dominguez Hills, Carson, CA, USA

## Abstract

As the world emerges from the COVID-19 pandemic, proactive planning for future pandemics is imperative to reduce morbidity and mortality. Central to this preparedness is the development of leaders equipped with the appropriate leadership frameworks and perspectives to navigate complex global health challenges. This commentary introduces an integrated definition of leadership and presents the Deliberate Proactive Leadership Framework as a model for fostering the skills and strategies necessary to address future crises effectively.

## 1 Narrative

The emergence of the COVID-19 pandemic in 2020 caught the entire world by surprise as the world was not prepared for such a novel disease with significant mortality and morbidity. Millions were affected across the nations of the world and many died. COVID-19, caused by the SARS-CoV-2 virus, affected virtually every country in the world as cases were confirmed in 222 countries, territories, and areas [1]. The global death toll from COVID-19 has exceeded 6.9 million people [2]. However, the World Health Organization states, “the true mortality is not well understood” and this figure may be a gross underestimation of the magnitude of the problems as the number of people who died was grossly underreported in some countries, and comprehensive documentation was stopped across the world while the virus was still mutating and killing people [[3], [4], [5]].

A lot has been written about the COVID-19 pandemic covering all aspects of the disease, the lessons learned, what we know today we wish we knew before the epidemic, why the epidemic spared Africans, and the inequities in the management of the pandemic [[6], [7], [8]]. The world was largely unprepared and several mistakes were made by governments, policymakers, and healthcare providers amplifying the impact of the pandemic [9]. Leaders at all levels faced the pandemic with the skill sets they had. Their mistakes led to several loss of lives, conspiracy theories, and mass resignation of healthcare workers [9,10]. Following the discovery of some of the mistakes, Bohmer et al., in 2020 documented 10 lessons from COVID field hospitals including the need to publicly acknowledge the uncertainty, focus on research, delegate authority, not to delay in making difficult (and unpopular decisions), shorten the feedback cycle, legitimize reversal (of decisions made), set expectations, include patients and their families (in decisions and planning processes), looking after the staff and internal customers, and being present to provide timely and needed guidance [11].

There will be other pandemics in the coming years. It is not an if, but a when. When this happens, will the world be ready, or caught again unprepared? To prevent what happened with COVID-19 from ever happening again, the world needs to plan for the next pandemic.

### 1.1 Establishing the right leaders

Leaders who will lead the next pandemic must see the full spectrum of leadership. During COVID-19, leaders did what they were used to which was inadequate for an unknown emerging disease like COVID-19. And because “healthcare is notorious for its slow pace and unreliability of innovation adoption” it took longer than necessary to translate discoveries from laboratories to the clinical practice to curtail COVID-19-mediated morbidities and mortalities [12]. Noted scholars like Whetten and Cameron defined leadership as “A temporary, dynamic condition that can be developed and demonstrated by any person willing to adopt a certain mindset and implement certain key skills and competencies” [13]. Earlier in 1995, Stoner saw leadership as “The process of directing and influencing the task-related activities of group members” [14] and McMahon et al., in 1992 defined leadership as the “Art of influencing, guiding and managing effectively.” [15] In 2000, Shortell and Kaluzny said that leadership is “the process through which an individual attempts to intentionally influence another individual or a group in order to accomplish a goal” [16]. As leadership is about making people better in the present and ensuring that the changes last, each of these definitions could be said to explain an aspect of leadership. However, none of these views integrated all aspects of leadership as proponents of these definitions saw leadership from one or more angles.

This partial perspective of leadership was the root cause of the problems the world faced with COVID-19 pandemic. People who were placed in leadership positions led using what they knew at that time which was merely a ‘part of the elephant’, as rather than a wholistic system approach, several focused on one or more aspects of the pandemic – the virus, mode of transmission, rate of spread, vaccine, economic impacts, etc. To ensure we build rounded leaders who will manage the next pandemic, we must establish the right leadership paradigm that sees leadership as “*the art and science of influencing, motivating, galvanizing, and inspiring people to effectively and efficiently utilize scarce resources to achieve health objectives that ensure sustainable health and well-being of the community across a defined area of jurisdiction”* [17,18]. This integrated definition of leadership sees leadership as an **ACT** – what is done to get what is needed. This includes influencing, motivating, galvanizing and inspiring people to use available resources effectively and efficiently to achieve identified goals. It also sees leadership as an **ART** – what is learned through practice, mentorship and formal and informal trainings; and a **SCIENCE** that evolves through studies, critical thinking, and experimentation; and could be passed unto others. When leaders see leadership from this integrated perspective, they do what is needed, learn to become better versions of themselves, and help build the next generation of leaders. This is the right leader to lead the next pandemics.

### 1.2 Using the right leadership framework

Leaders who will successfully lead the next pandemics must have an integrated framework. Over the years, exceptional leaders have developed effective leadership frameworks that have moved the practice of leadership forward. These include Kurt Lewin and his colleagues framework that categorized leadership into three main styles: autocratic, democratic, and laissez-faire, Weber's bureaucratic leadership style, and Robert K. Greenleaf servant leadership [19]. Others include James MacGregor Burns' transformational and transactional leadership styles, Daniel Goleman's six leadership styles (coercive, authoritative, affiliative, democratic, pacesetting, and coaching), and Fred Fiedler's contingency leadership. In addition, we have Robert J House's path-goal leadership style, David McClelland's competency-based leadership, Paul Hersey and Ken Blanchard's situational leadership, Bill George's authentic leadership, James Rest's ethical leadership, amongst several others [19]. Each of these styles of leadership helps a leader solve a particular kind of issue but does not serve them in every situation. Leaders during COVID-19 era used one or more of these known styles depending on their training, exposure and environment to address unique aspects of leadership. Leaders who will successfully lead the next pandemic must use an integrated leadership framework such as the Deliberate Proactive (DP) Leadership Style [17,18]. The DP leadership style integrates all leadership styles into one, and emphasizes the need for leaders to Think, Plan, Prepare, Provide, and Communicate Ahead. DP leaders use the following constructs - Self-Efficacy, Self-Control, Self-Empowerment, and Self-Encouragement leading to Self-Fulfillment [17].

DP leaders have a vision, work with a strategy, have a people-oriented mindset, use defined processes, and are result-oriented and timeliness-minded because they know that time cannot be stopped, stretched, stockpiled, or recovered, but is a very limited resource that once lost cannot be recovered. In addition, DP leaders take full responsibility for their actions, results, and failures; and develop succession plans to create a new generation of leaders as they build and develop others to do better, achieve more, and become great leaders.

## 2 Conclusion

The receding COVID pandemic infected over 676,609,955, led to the death of over 6,881,955 and resulted in the administration of over 13,338,833,198 vaccinations within recorded time [2]. Despite the massive investments made, the world was negatively impacted in several ways. Some of these negative impacts could have been mitigated if leaders used an integrated system-based approach to manage the pandemic from the very beginning of the epidemic.

Leaders who will successfully manage the next natural or man-made pandemic must understand leadership as an art, act, and science of influencing, motivating, galvanizing, and inspiring people to effectively and efficiently utilize scarce resources to achieve health objectives that ensure sustainable health and well-being of the community [17,18]. They must also lead using the Deliberate Proactive (DP) leadership framework to Think, Plan, Prepare, Provide and Communicate Ahead towards organizational success, thereby minimizing mistakes and improving outcomes.

## Declaration of competing interest

I have nothing to declare.
